# Detection of circulating genetically abnormal cells using 4-color fluorescence in situ hybridization for the early detection of lung cancer

**DOI:** 10.1007/s00432-021-03517-6

**Published:** 2021-02-06

**Authors:** Mingxiang Feng, Xin Ye, Baishen Chen, Juncheng Zhang, Miao Lin, Haining Zhou, Meng Huang, Yanci Chen, Yunhe Zhu, Botao Xiao, Chuoji Huang, Ruth L. Katz, Chunxue Bai

**Affiliations:** 1 Division of Thoracic Surgery, Zhongshan Hospital, Fudan University, Shanghai, China; 2 Zhuhai Sanmed Biotech. Ltd, Zhuhai, China; 3 Joint Research Center of Liquid Biopsy in Guangdong, Hongkong and Macao, Zhuhai, China; 4 Department of Thoracic surgery, Respiratory Center of Suining Central Hospital, An Affiliated Hospital of Chongqing Medical University, An Affiliated Hospital of North Sichuan Medical College, Suining, Sichuan China; 5 Department of Thoracic Surgery, Sun Yat-Sen Memorial Hospital of Sun Yat-Sen University, Guangzhou, China; 6 School of Biology and Biological Engineering, South China University of Technology, Guangzhou, China; 7 Department of Pathology, The University of Texas MD Anderson Cancer Center, Houston, TX USA

**Keywords:** Non-small cell lung cancer, Early detection of cancer, Early diagnosis, Circulating genetically abnormal cells, Fluorescence in situ hybridization

## Abstract

**Purpose:**

Available biomarkers lack sensitivity for an early lung cancer. Circulating genetically abnormal cells (CACs) occur early in tumorigenesis. To determine the diagnostic value of CACs in blood detected by 4-color fluorescence in situ hybridization (FISH) for lung cancer.

**Methods:**

This was a prospective study of patients with pulmonary nodules ≤ 30 mm detected between 10/2019 and 01/2020 at four tertiary hospitals in China. All patients underwent a pathological examination of lung nodules found by imaging and were grouped as malignant and benign. CACs were detected by 4-color FISH. Patients were divided into the training and validation cohorts. Receiver operating characteristics analysis was used to analyze the diagnosis value of CACs.

**Results:**

A total of 205 participants were enrolled. Using a cut-off value of ≥ 3, blood CACs achieved areas under the curve (AUCs) of 0.887, 0.823, and 0.823 for lung cancer in the training and validation cohorts, and all patients, respectively. CACs had high diagnostic values across all tumor sizes and imaging lesion types. CACs were decreased after surgery (median, 4 vs. 1, *P* < 0.001) in the validation set. The CAC status between blood and tissues was highly consistent (kappa = 0.909, *P* < 0.001). The AUC of CAC (0.823) was higher than that of CEA (0.478), SCC (0.516), NSE (0.506), ProGRP (0.519), and CYFRA21-1 (0.535) (all *P* < 0.001).

**Conclusion:**

CACs might have a high value for the early diagnosis of lung cancer. These findings might need to be validated in future studies. Evidence suggested homology in genetic aberrations between the CACs and the tumor cells.

**Supplementary Information:**

The online version contains supplementary material available at 10.1007/s00432-021-03517-6.

## Introduction

Lung cancer is the leading cause of mortality globally, especially in smokers and the elderly (NCCN Clinical Practice Guidelines in Oncology (NCCN Guidelines). Non-Small Cell Lung Cancer 2020; Novello et al. 2016). There were an estimated 2,093,876 new cases of lung cancer worldwide in 2018, with an annual age-standardized incidence of 31.5/100,000 in men and 14.6/100,000 in women (Bray et al. 2018). The National Comprehensive Cancer Network (NCCN), the CHEST guideline and expert panel report, and the US Preventive Services Task Force recommended low-dose computed tomography (LDCT) screening for people aged 55–74, currently smoking or with ≥ 30 pack-year history of smoking, and past smokers for < 15 years (Mazzone et al. 2018; Moyer and Force 2014; NCCN Clinical Practice Guidelines in Oncology (NCCN Guidelines). Lung Cancer Screening 2019), and screening can be considered in individuals ≥ 50 years of age, a ≥ 20 pack-year history of smoking, and additional risk factors (including personal history of cancer or lung disease, family history of lung cancer, radon exposure, or relevant occupational exposure) that increases the risk of lung cancer to ≥ 1.3% (not including second-hand smoke exposure) (Mazzone et al. 2018; Moyer and Force 2014; NCCN Clinical Practice Guidelines in Oncology (NCCN Guidelines). Lung Cancer Screening. 2019). Early diagnosis is paramount for prognosis, since the five-year survival rate is 56% for patients with localized disease, 30% for those with regional disease, but only 5% for individuals distant disease (NCCN Clinical Practice Guidelines in Oncology (NCCN Guidelines). Lung Cancer Screening 2019; NCCN Clinical Practice Guidelines in Oncology (NCCN Guidelines). Non-Small Cell Lung Cancer 2020).

The current early detection methods for lung cancer are not sufficient. Indeed, available biomarkers (like carcinoembryonic antigen) and circulating nucleic acids have a low sensitivity (O’Flaherty et al. 2017; Plaks et al. 2013; Sorber et al. 2017). Plain X-ray, computed tomography (CT), and positron-emission tomography (PET)/CT have relatively high rates of false-positive and have low sensitivity for tumors < 10 mm and pure ground-glass nodules (National Lung Screening Trial Research et al. 2011; Wisnivesky et al. 2006; Yamagami et al. 2004; Yamauchi et al. 2011). Importantly, biopsy is an invasive diagnosis method and is associated with possible complications like infection and pneumothorax.

Genetic abnormalities in tumor suppressor genes and proto-oncogene are common in lung cancer (Hirsch et al. 2001; Zochbauer-Muller et al. 2002), but cannot be used for the screening of lung cancer because of the low yield of cancer cells in the blood, tumor heterogeneity, and unknown imminency between the detection of the abnormality and actual malignant transformation (Romeo et al. 2003). Cancer cells are often unable to maintain chromosome numbers and structures because of rapid uncontrolled growth and division (Romeo et al. 2003). Chromosomal aberrations, including rearrangements and aneusomy, are frequently found in early lung cancer (Haruki et al. 2001; Schenk et al. 1997; Taguchi et al. 1996). Importantly, a study of seven lung cancer specimens showed that chromosomal instability is found both in 8.5% and 59% of the premalignant and malignant lesions, respectively, within the same patients (Zojer et al. 2000), indicating that specific chromosomal aberrations occur in the early stage of tumorigenesis (Romeo et al. 2003). Circulating genetically abnormal cells (CACs; i.e., cells that carry chromosomal instability) occur early in tumorigenesis and CACs can be detected from the blood (Katz et al. 2010, 2020). Katz et al. developed a 4-color fluorescence in situ hybridization (FISH) assay to identify CACs from peripheral blood of lung cancer patients using chromosomes 3 and 10 (probes for 3p22.1/3q29 (196F4) and 10q22.3/CEP10) (Katz et al. 2010). These abnormalities have previously been shown to commonly occur in lung cancer samples by the comparative genomic hybridization analysis (Jiang et al. 2004).

The study aimed to evaluate the diagnostic value of CACs detected by 4-color FISH for lung cancer, as well as to examine the genetic abnormalities between CACs and tumor cells. The results could be a novel sensitive and specific biomarker for the early detection of the disease.

## Materials and methods

### Study design and participants

This was a prospective study of patients with pulmonary nodules detected between October 2019 and January 2020 at the Zhongshan Hospital Affiliated to Fudan University, the Second Hospital Affiliated to Zhongshan University (Sun Yat-Sen Memorial Hospital), Suining Central Hospital, and Shanghai Chest Hospital. The study has been approved by the ethics committee of Zhongshan Hospital Affiliated to Fudan University (b2019-185r) and by the ethics review committees of the three other hospitals. Written inform consent was provided by all participants before the study.

The inclusion criteria were: (1) single or multiple pulmonary nodules ≤ 30 mm detected by CT or LDCT within the past 6 months; (2) ≥ 18 years of age; and (3) planned to undergo non-surgical biopsy or surgical resection of the pulmonary nodules and histopathological examination. The exclusion criteria were: (1) lactating, pregnant, or preparing pregnant women; (2) severe heart, lung, liver, or kidney dysfunction or mental disorders; (3) previous clinical therapeutic interventions related to lung cancer, such as surgery, radiotherapy, chemotherapy, targeted treatment, or immunotherapy; (4) sampling problem that could not meet the requirements for histopathological examination; or (5) history of a malignant tumor within 5 years.

### Grouping

CAC detection was performed for all participants within 5 days preoperatively, and within 5 days postoperatively for some of them. We enrolled the participants without postoperative blood collection as the training set and those with postoperative blood collection as the validation set.

### CAC detection

Peripheral blood (10 ml) and tissue samples were collected preoperatively and postoperatively for CAC detection. Chromosome 3 and 10 [probes for 3p22.1/3q29 (196F4) and 10q22.3/CEP10] abnormalities of peripheral blood mononuclear cells (PBMCs) of the pulmonary nodule population were qualitatively detected using the Mononuclear Cell Chromosomal Abnormality Detection Kit (Zhuhai Sanmed Biotechnology Ltd.). The assay was performed according to manufacturer’s manual and was described in previous publications (cite our own papers). In brief, PBMCs were enriched via Ficoll density gradient and deposited to microscope glass slides by Cytospin system (Thermo Fisher). The cells were subsequently fixed for 4-color FISH (Katz et al. 2010, 2020; Yendamuri et al. 2008) or storage at − 20 °C. Cell nuclei were stained with 4′, 6-diamidino-2-phenylindole (DAPI).

The FISH samples were digitalized by the Duet System (Allegro plus, BioView Ltd.) to visualize the chromosomal targets in Chr. 3 and 10. Signal distribution in each cell was enumerated to identify chromosome loci gain or loss. Cells with polysomy in at least two different fluorescence channels were characterized as CACs.

### Biomarkers

Peripheral blood samples were taken from each participant in 3-ml anticoagulant tubes for the measurement of the carcinoembryonic antigen (CEA), progastrin-releasing protein (ProGRP), squamous cell carcinoma antigen (SCC), and CYFRA21-1 levels. On the same day as collection, the tumor biomarkers were measured using electrochemiluminescence immunoassays (ECLIA) on a Roche Elecsys E170 analyzer (Roche Diagnostics, Switzerland).

### Data collection

The characteristics of the participants (age, sex, and smoking history) were collected. The diagnosis of the nodules was classified into malignant (lung squamous cell carcinoma, invasive adenocarcinoma, micro-invasive adenocarcinoma, and malignant others) and benign (benign tumors, infection/inflammatory lesions, and benign others). The largest nodule size and the largest nodule type for each patient were recorded. LDCT examination was performed by the 64-section multidetector CT machine (Siemens, Erlangen, Germany).

### Statistical analysis

Continuous data with a normal distribution were presented as means ± standard deviation and analyzed using the independent sample *t*-test. Continuous data with a skewed distribution were presented as medians (interquartile ranges) and analyzed using the Mann–Whitney *U* test. Categorical data were expressed as *n* (%) and analyzed using the chi-square test or Fisher’s exact probability test. Receiver operating characteristic (ROC) curves were used to identify the cut-off value for CACs, the area under the curve (AUC), sensitivity, specificity, and other indicators. The numbers of postoperative and preoperative CACs in the validation set were tested using the Wilcoxon signed-rank sum test. The consistency of CACs in blood and tissue was tested by the Kappa test. All analyses were performed using SPSS 22.0 (IBM, Armonk, NY, USA). Two-sided *P* values < 0.05 were considered statistically significant.

## Results

### Baseline information

A total of 205 participants were enrolled (112 in the training set and 93 in the validation set) (Table 1). There were 97 (47.3%) males. The median age was 62 (54–67) years. Among the 205 participants, 168 (82.0%) had malignant lesions. The median longest diameter of the nodules was 18 (12–24) mm. Seventy participants (34.2%) had pure ground-glass lesions, 96 (46.8%) had solid lesions, and 39 (19.0%) had mixed lesions. CACs were counted from the participants’ blood before and after the operation; the median preoperative and postoperative CAC counts were 4 (2–7) and 1 (0–5), respectively. There were no significant differences between the two sets (all P > 0.05).

**Table 1:** Baseline characteristics of the patients

	Total *n* = 205	Training set *n* = 112	Validation set *n* = 93	*P*
Sex, *n* (%)				0.573
Male	97 (47.3%)	55 (49.1%)	42 (45.2%)	
Female	108 (52.7%)	57 (50.9%)	51 (54.8%)	
Age, median (IQR)	62 (54, 67)	62 (55,68)	62 (52,66)	0.246
Smoking history, *n* (%)	51 (24.9%)	28 (25.0%)	23 (24.7%)	0.965
Benign and malignant, *n* (%)				0.310
Benign	37 (18.1%)	23 (20.5%)	14 (15.1%)	0.692
Benign tumor	11 (5.4%)	8 (7.1%)	3 (3.2%)	
Infection/inflammatory lesions	22 (10.7%)	13 (11.6%)	9 (9.7%)	
Other	4 (2.0%)	2 (1.8%)	2 (2.2%)	
Malignant	168 (82.0%)	89 (79.5%)	79 (85.0%)	0.113
Squamous cell carcinoma of lung	8 (3.9%)	2 (1.8%)	6 (6.5%)	
Invasive adenocarcinoma	84 (41.0%)	44 (39.3%)	40 (43.0%)	
Microinvasive adenocarcinoma	72 (35.1%)	39 (34.8%)	33 (35.5%)	
Other	4 (2.0%)	4 (3.6%)	0	
Maximum nodule size, median (IQR)	18 (12, 24)	18 (13, 23)	19 (11, 25)	0.560
Maximum nodule type, n (%)				0.074
Pure ground glass type	70 (34.2%)	42 (37.5%)	28 (30.1%)	
Mixed	39 (19.0%)	15 (13.4%)	24 (25.8%)	
Solid	96 (46.8%)	55 (49.1%)	41 (44.1%)	
Preoperative CAC, median (IQR)	4 (2, 7)	4 (2, 6)	4 (3, 7)	0.893
Postoperative CAC, median (IQR)	1 (0, 5)	NA	1 (0, 5)	NA

*IQR* interquartile range, *CAC* genetically abnormal cells

### ROC analysis of CACs for the diagnosis of lung cancer

**Fig. 1:**
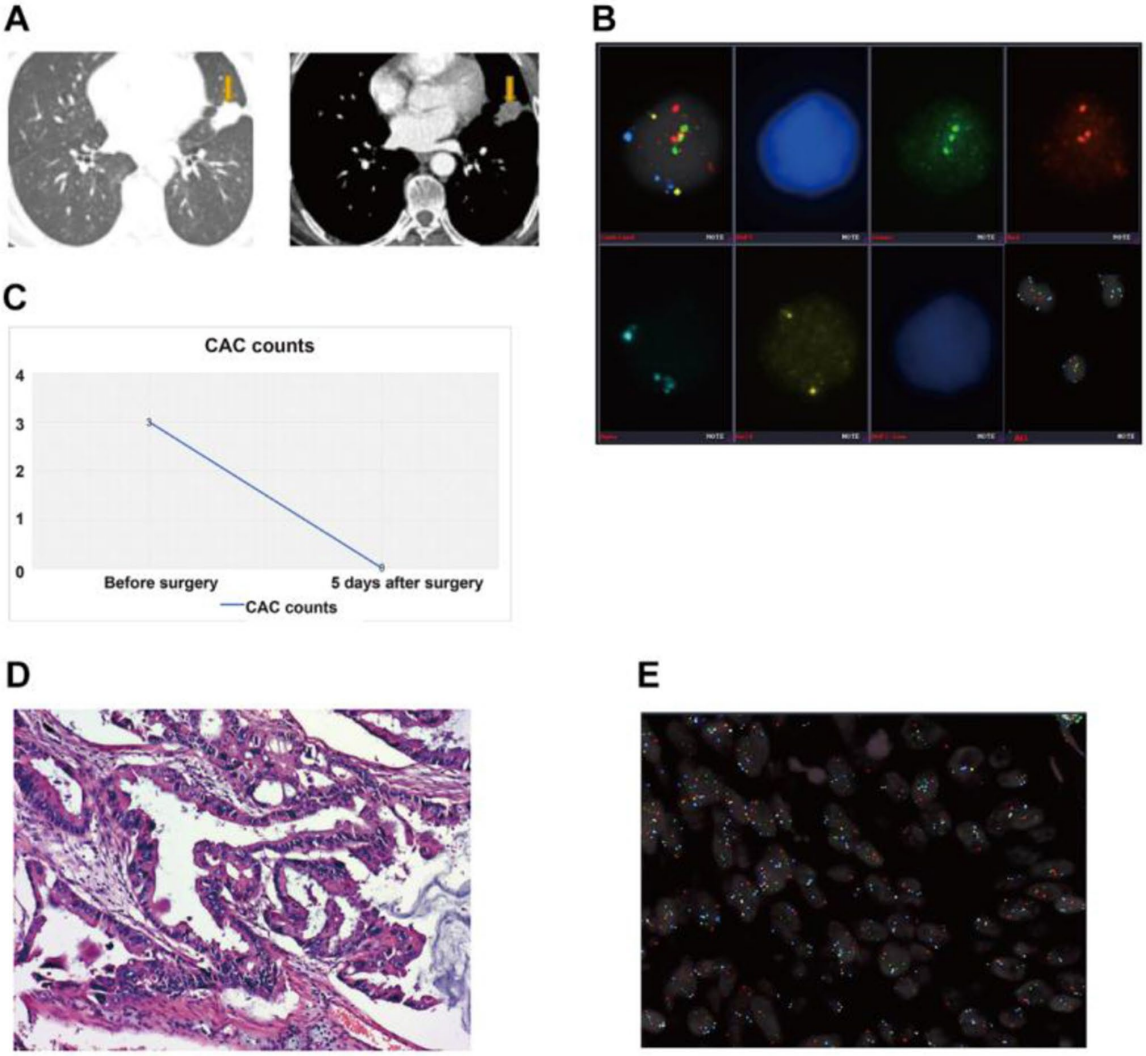
A typical case. **a** Preoperative computed tomography scans. **b** CAC schematic diagram. Cells with 4ʹ, 6-diamidino-2-phenylindole (DAPI) staining (original magnification × 100). The combined images of CACs show the polysomy/gain of 3p22.1 (red), polysomy/gain of 3q29 (green), and CEP10 (aqua), whereas 10q22.3 (gold, two copies) was diploid. Genetic abnormalities were identified using a 4-color cocktail of FISH probes on a BioView Duet-3 instrument (original magnification × 400): three red signals consistent with three copies of 3p22.1; three aqua signals representing three copies of CEP10; three green signals representing three copies of 3q29; and two gold signals consistent with two copies of 10q22.3. **c** Three CACs were found by a 4-color cocktail of FISH probes on a BioView Duet-3 instrument. **d** Pathological HE revealed adenocarcinoma of the lung. **e** Chromosomal abnormalities in the tissue

Figure 1 shows a typical case of a patient with a large solid nodule in the lung and positive CAC results. Preoperative CT revealed a solid nodule (Fig. 1a). CACs were analyzed by 4-color fluorescence in situ hybridization (Fig. 1b). The CEP10 is diploid and it has a split/diffused signal. The combined images of CACs show polysomy/gain of 3p22.1 (red), polysomy/gain of 3q29 (green), whereas 10q22.3 (gold, two copies) and CEP10 (aqua) was diploid (Fig. 1c). The participant had 3 CACs before surgery and 0 after surgery (Fig. 1d). Histopathological examination revealed adenocarcinoma of the lung. The chromosomal abnormalities were also found in the tissue specimens (Fig. 1e).

**Fig. 2:**
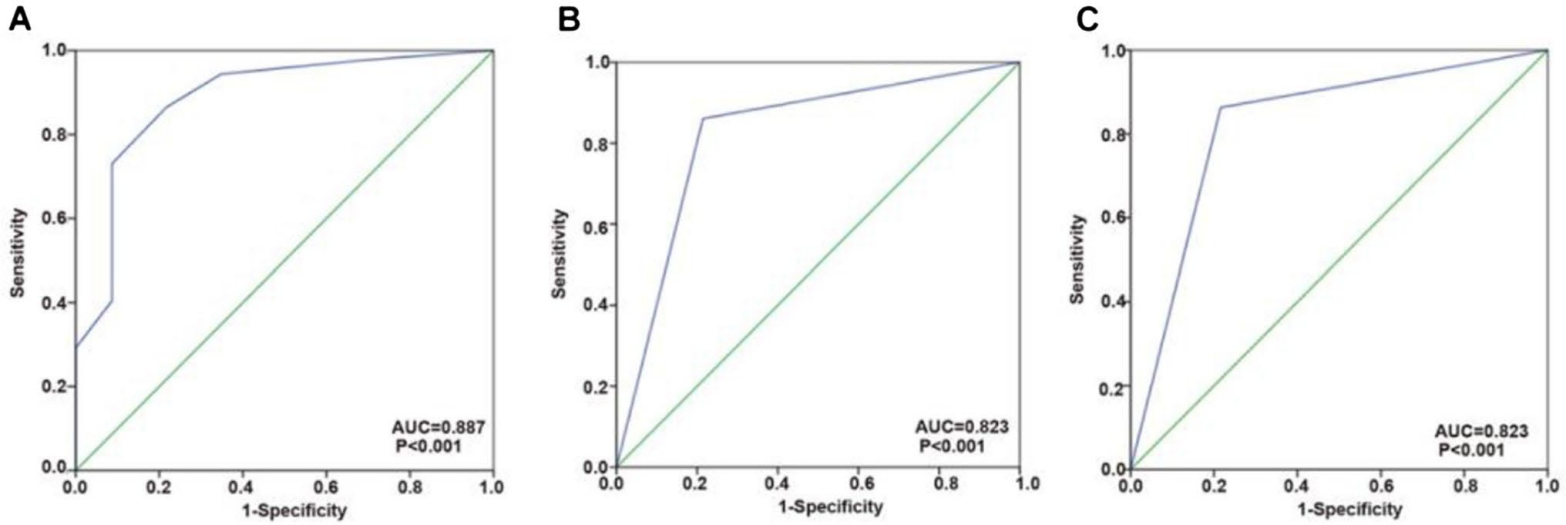
Receiver operating characteristics analysis of genetically abnormal cells (CAC) for non-small cell lung cancer. **a** Training set. Using a cut-off value of ≥ 3 CAC achieved 86.5% sensitivity, 78.3% specificity, the area under the curve (AUC) of 0.887, 93.9% positive predictive value (PPV), 60.0% negative predictive value (NPV), and 3.98 positive likelihood-ratio (PLR). **b** Validation set. Using a cut-off value of ≥ 3 CAC achieved 86.1% sensitivity, 78.6% specificity, AUC of 0.823, 95.8% PPV, 50.0% NPV, and 4.02 PLR. **c** All patients. Using a cut-off value of ≥ 3 CAC achieved 86.3% sensitivity, 78.4% specificity, AUC of 0.823, 94.8% PPV, 55.8% NPV, and 3.99 PLR

We achieved an 86.5% sensitivity rate, a 78.3% specificity, an AUC of 0.887, a 93.9% positive predictive value (PPV), a 60.0% negative predictive value (NPV), and the 3.98 positive likelihood ratio (PLR) in the training set, when the cut-off value is set as ≥ 3 CAC (Fig. 2). In the validation set, using the same CAC cut-off value, we observed 86.1% sensitivity, 78.6% specificity, 0.823 AUC, 95.8% PPV, 50.0% NPV, and 4.02 PLR (Fig. 2b). In all participants, using a cut-off value of ≥ 3 CACs, 86.3% sensitivity, 78.4% specificity, 0.823 AUC, 94.8% PPV, 55.8% NPV, and 3.99 PLR were obtained (Fig. 2c). Supplementary Table 1 shows the sensitivity and specificity values according to different CAC cut-off levels. It should be noted that the three data sets of specimens yielded similar results, suggesting that the training and validation sets were consistent.

### CAC diagnostic power in different nodule sizes

The participants were divided according to their lesion size. In participants with a lesion of 0–9 mm, using a cut-off value of ≥ 3, CAC achieved 84.2% sensitivity, 85.7% specificity, AUC of 0.850, 94.1% PPV, 66.7% NPV, and 5.90 PLR (Supplementary Fig. 1a). In participants with a lesion of 10–29 mm, CAC achieved 85.5% sensitivity, 74.1% specificity, AUC of 0.798, 94.4% PPV, 50.0% NPV, and 3.30 PLR (Supplementary Fig. 1b). In participants with a lesion of 30 mm, CAC achieved 100% sensitivity, 100% specificity, AUC of 1.000, 100% PPV, 100% NPV, and not-applicable PLR (Supplementary Fig. 1c). Those results indicate that the tumor size has minimal or no impact on the diagnostic value of CACs.

### CAC diagnostic power in different nodule types

The participants were grouped according to the type of lung lesion at imaging. In participants with a pure ground glass, solid, and mixed lesions and using a cut-off value of ≥ 3, CAC achieved 82.0%/90.4%/85.3% sensitivity, 77.8%/73.9%/100% specificity, AUC of 0.799/0.822/0.926, 96.2%/91.7%/100% PPV, 38.9%/70.8%/50.0% NPV, and 3.69/3.47/NA PLR (Supplementary Fig. 2). Those results indicate that the type of radiological lesion has no impact on the diagnostic value of CACs.

### Comparison of CACs in patients with single or multiple nodules

26 subjects had multiple nodules and the other 179 had a single nodule. The median number of CACs in patients with single nodule was similar to that of patients with multiple nodules (4 vs. 4).

### Correlation between blood and corresponding lung cancer tissue

78 patients’ peripheral blood and tumor tissues, who underwent surgical resection of their lung tumors, were obtained in paired sets. The same set of 4-color FISH probes was used in both the blood and tumor specimens. We observed a significant correlation between four genetical abnormalities in PBMCs and corresponding biomarkers in the tumor specimens. Table 2 shows the postoperative and preoperative CAC counts in the validation set. The CAC count was significantly decreased after the surgery (median, 4 vs. 1, *P* < 0.001). Table 3 shows that the genetic abnormalities between CACs and tumor cells were highly consistent (kappa = 0.909, *P* < 0.001).

**Table 2:** Comparison of the numbers of preoperative and postoperative CACs

	Preoperative CAC	Postoperative CAC	*P*
Median (IQR)	4 (3, 7)	1 (0, 5)	< 0.001

*IQR* interquartile range, *CAC* genetically abnormal cells

**Table 3:** Comparison in chromosomal abnormalities in blood and tissue

	Positive tissue chromosome	Negative tissue chromosome	Kappa	*P*
Blood positive (CAC ≥ 3)	53	3	0.909	< 0.001
Blood negative (CAC < 3)	0	22		

*CAC* genetically abnormal cells

Of the 78 subjects, 63 were diagnosed with malignant tissues in histopathological examinations. Of these 63 subjects, 52 (82.54%) had chromosome abnormalities detected in the tissue test, while none of the 15 subjects with benign tissues had chromosome abnormalities in the tissue test.

### Sensitivity and specificity of common biomarkers

The diagnostic value of different biomarkers was assessed and compared with CACs (Table 4). The AUC of CAC (0.823) was higher than that of CEA (0.478), SCC (0.516), NSE (0.506), ProGRP (0.519), and CYFRA21-1 (0.535) (all *P* < 0.001).

**Table 4:** Sensitivity and specificity of common biomarkers

Biomarker	AUC	*P*	95% CI	Sensitivity	Specificity	Comparison with AUC of CAC
CEA	0.478	0.684	(0.372, 0.585)	0.151	0.806	< 0.001
SCC	0.516	0.775	(0.408, 0.624)	0.032	1.000	< 0.001
NSE	0.506	0.915	(0.399, 0.613)	0.071	0.941	< 0.001
Pro-GRP	0.519	0.794	(0.383, 0.654)	0.037	1.000	< 0.001
CYFRA21-1	0.535	0.522	(0.432, 0.637)	0.184	0.886	< 0.001
CAC	0.823	< 0.001	(0.741, 0.906)	0.863	0.784	

*AUC* area under the curve, *CI* confidence interval, *IQR* interquartile range, *CAC* genetically abnormal cells, *CEA* carcinoembryonic antigen, *SCC* squamous cell carcinoma antigen, *NSE* neuron-specific enolase, *pro-GRP* pro-gastrin releasing peptide

## Discussion

It has been well-documented that the commonly available serum tumor biomarkers provide a little diagnostic value for early lung cancer diagnosis (National Lung Screening Trial Research et al. 2011; O’Flaherty et al. 2017; Plaks et al. 2013; Sorber et al. 2017; Wisnivesky et al. 2006; Yamagami et al. 2004; Yamauchi et al. 2011). CACs occur early in tumorigenesis and might be of use as a biomarker for lung cancer (Katz et al. 2010, 2020). The results of this study indicate that CACs have a high value for the early diagnosis of lung cancer. This will have to be validated in future studies as an early screening tool for lung cancer. Nevertheless, the results could be a novel sensitive and specific biomarker for the early detection of the disease. In many studies on the early diagnosis of lung cancer, the control group includes healthy people (without nodules). The disadvantage of this control group is the lack of histopathological results, and the actual absence of lung cancer cannot be confirmed. Therefore, in the present study, the control group included patients with benign lung nodules proven pathologically.

In this study, CACs show a high diagnostic value for tumors of all sizes, even for tumors of 0–10 mm. This is of particular interest in a screening context because PET-CT has low sensitivity for tumors of 0–10 mm (sensitivity of 50% for lesions < 10 mm or 17% for lesions < 8 mm) (Evangelista et al. 2012; Kim et al. 2007), and PET-CT is not indicated for lesions < 8 mm or < 10 mm (Cancer du poumon, Bilan initial, Collection Recommandations et référentiels 2011; Patel et al. 2013a, b). In the case of misdiagnosis, even though the patient could be followed up and get diagnosed in the next scanning, he/she could miss the best treatment window and allow the tumor to further develop and metastasize (NCCN Clinical Practice Guidelines in Oncology (NCCN Guidelines); Lung Cancer Screening 2019; NCCN Clinical Practice Guidelines in Oncology (NCCN Guidelines). Non-Small Cell Lung Cancer 2020). However, while other studies have suggested that the CAC number might increase as the tumor progresses and be related to tumor prognosis (Katz et al. 2020), we did not find a relationship with tumor size in this study. This could be because all the patients had tumors of less than 30 mm. Further study of the patients with a longer follow-up might show the CAC numbers increase if the disease progresses. The type of lesion had a minimal influence on the diagnostic value. This study is the first to analyze the diagnostic value of CACs for lung cancer across lesion size, imaging lesion type, and comparing its performance with common tumor biomarkers. The results showed high AUCs for all included lesions ≤ 30 mm, which are the lesions commonly found during early screening and considered benign (NCCN Clinical Practice Guidelines in Oncology (NCCN Guidelines). Lung Cancer Screening 2019).

This method of investigating CACs has some advantages over conventional chromosome testing of cells, which is performed by FISH-staining the tissues. Staining cells with chromosome abnormalities in peripheral blood involves liquid biopsy, which enables multiple, repeated and non-invasive evaluation of chromosome abnormalities in patients. Moreover, our system enables the automatic recognition and automatic counting of blood cells with chromosome abnormalities, which reduces the impact of observer bias. We also showed that the genetic abnormalities in the CACs were similar to those found in their respective tumors. This is supported by previous studies, in which CACs had similar characteristics to those of the primary tumor cells (Katz et al. 2010; Pailler et al. 2015). This homology might be an indication that the CACs found in peripheral blood might be tumor cells that detached themselves from the primary tumor and entered circulation in order to seed novel metastases. Besides, after the surgery, the CACs were significantly reduced, which supports the view that there was homology in genetic aberrations between the CACs and the tumor cells.

The numbers of CACs might very well represent the lung lesion and could be used for the early diagnosis of lung cancer. Indeed, more aggressive tumors will show less cell cohesion, leading to more CACs with the potential to seed metastases (Yendamuri et al. 2008). Of note, CACs can be found in premalignant lesions, and malignant lesions since the appearance of cytogenetic abnormalities that occur early in tumorigenesis (Zojer et al. 2000), highlighting their value for the early detection of lung cancer.

Multiple biomarkers in blood are used for tumor liquid biopsies, yet none has been extensively validated and utilized in clinical settings (O'Flaherty et al. 2017; Plaks et al. 2013; Sorber et al. 2017). The sensitivity and specificity of ctDNA for early lung cancer were 53.8% and 47.3%, respectively (Chen et al. 2016). Regarding CTCs detected using the Veridex CellSearch system, which is based on an anti-EpCAM antibody, the sensitivity for early lung cancer was only 19.3% (Tanaka et al. 2009). In comparison, the present study showed a sensitivity of 86% to detect lung cancer in all participants. Nevertheless, this conclusion must be taken with caution as those different biomarkers were not assessed head-to-head in the same patients. Future studies should include CACs, CTCs, and tumor DNA, and those should be tested in healthy controls for the validation to ensure that those tests are negative in patients without cancer.

More traditional biomarkers like CEA, ProGRP, SCC, NSE, and CYFRA21-1 are commonly used for the diagnosis and management indicators of lung cancer (Mishra and Verma 2010; NCCN Clinical Practice Guidelines in Oncology (NCCN Guidelines). Non-Small Cell Lung Cancer 2020; Novello et al. 2016), but they are not designed for the early screening of lung cancer due to low sensitivity (NCCN Clinical Practice Guidelines in Oncology (NCCN Guidelines). Lung Cancer Screening 2019; Neal et al. 2015; Vargas and Harris 2016). In the present study, the AUC and sensitivity of CACs for early lung cancer were higher than any of those five biomarkers, suggesting that CAC could be a sensitive marker for lung cancer early diagnosis working in conjunction with LDCT.

Despite its strengths, there are several limitations in this study. For instance, we only assessed a relatively small cohort with high disease prevalence, which may not be sufficient to establish reliable correlations between CACs and the clinicopathological characteristics of the patients. Nevertheless, a strength of the present study is that all participants had a pathological diagnosis, even the benign nodules. Many previous studies included patients with tuberculosis or chronic inflammation, and they rarely included those with benign tumors and granuloma. Additional studies with larger cohorts would be needed to guarantee the robustness of the ROC analysis and identify most powerful threshold of CACs for lung cancer early diagnosis. Another limitation was that other popular liquid biopsies biomarkers, such as ctDNA and CTC, were not parallelly analyzed. Notwithstanding these limitations, the study findings underscore an interesting biological process during lung cancerogenesis and identification of a novel biomarker for lung cancer early diagnosis.

In conclusion, CACs could be a promising biomarker for the early diagnosis of lung cancer. In a screening context, implementing such a diagnostic tool may benefit lung cancer patients with detection at an early stage and improve prognosis. Our study also suggests that there may be a high homology in genetic abnormalities between the CACs and the tumor cells in cancer tissue.

## Supplementary Information

Below is the link to the electronic supplementary material.

Supplementary file 1 (DOC 793 KB)

## Data Availability

The datasets used and/or analyzed during the current study are available from the corresponding author on reasonable request.

## Abbreviations

NCCN: National Comprehensive Cancer Network
LDCT: Low-dose computed tomography
CT: Computed tomography
PET: Positron-emission tomography
CACs: Circulating genetically abnormal cells
FISH: Fluorescence in situ hybridization
CEA: Carcinoembryonic antigen
ProGRP: Progastrin-releasing protein
SCC: Squamous cell carcinoma antigen
ECLIA: Electrochemiluminescence immunoassays
ROC: Receiver operating characteristic
AUC: Area under the curve
