# Supplementary material for: Detection of circulating genetically abnormal cells using 4-color fluorescence in situ hybridization for the early detection of lung cancer
Source: J Cancer Res Clin Oncol. 2021 Feb 6;147(8):2397–405. doi: 10.1007/s00432-021-03517-6 (PMC8236478; doi:10.1007/s00432-021-03517-6)

**Supplementary Table 1.** Cut-off value, sensitivity, and specificity under different conditions

|  | Cut-off | Sensitivity | Specificity |
| --- | --- | --- | --- |
| Training set |  |  |  |
| Maximum sensitivity | 0.5 | 0.978 | 0.304 |
| Maximum Youden index | 2.5 | 0.865 | 0.783 |
| Maximum specificity | 3.5 | 0.730 | 0.913 |
| Validation set |  |  |  |
| Maximum sensitivity | 0.5 | 0.975 | 0.286 |
| Maximum Youden index | 2.5 | 0.861 | 0.786 |
| Maximum specificity | 7.5 | 0.253 | 0.929 |
| Total |  |  |  |
| Maximum sensitivity | 0.5 | 0.976 | 0.297 |
| Maximum Youden index | 2.5 | 0.863 | 0.784 |
| Maximum specificity | 7.5 | 0.250 | 0.973 |

**Supplementary Figure 1. Receiver operating characteristics analysis of genetically abnormal cells (CAC) for non-small cell lung cancer according to tumor size. (A)** 0-9 mm. Using a cut-off value of ≥3 CAC achieved 84.2% sensitivity, 85.7% specificity, the area under the curve (AUC) of 0.850, 94.1% positive predictive value (PPV), 66.7% negative predictive value (NPV), and 5.90 positive likelihood ratio (PLR). **(B)** 10-29 mm. Using a cut-off value of ≥3 CAC achieved 85.5% sensitivity, 74.1% specificity, AUC of 0.798, 94.4% PPV, 50.0% NPV, and 3.30 PLR. **(C)** 30 mm. Using a cut-off value of ≥3 CAC achieved 100% sensitivity, 100% specificity, AUC of 1.000, 100% PPV, 100% NPV, and not-applicable PLR.


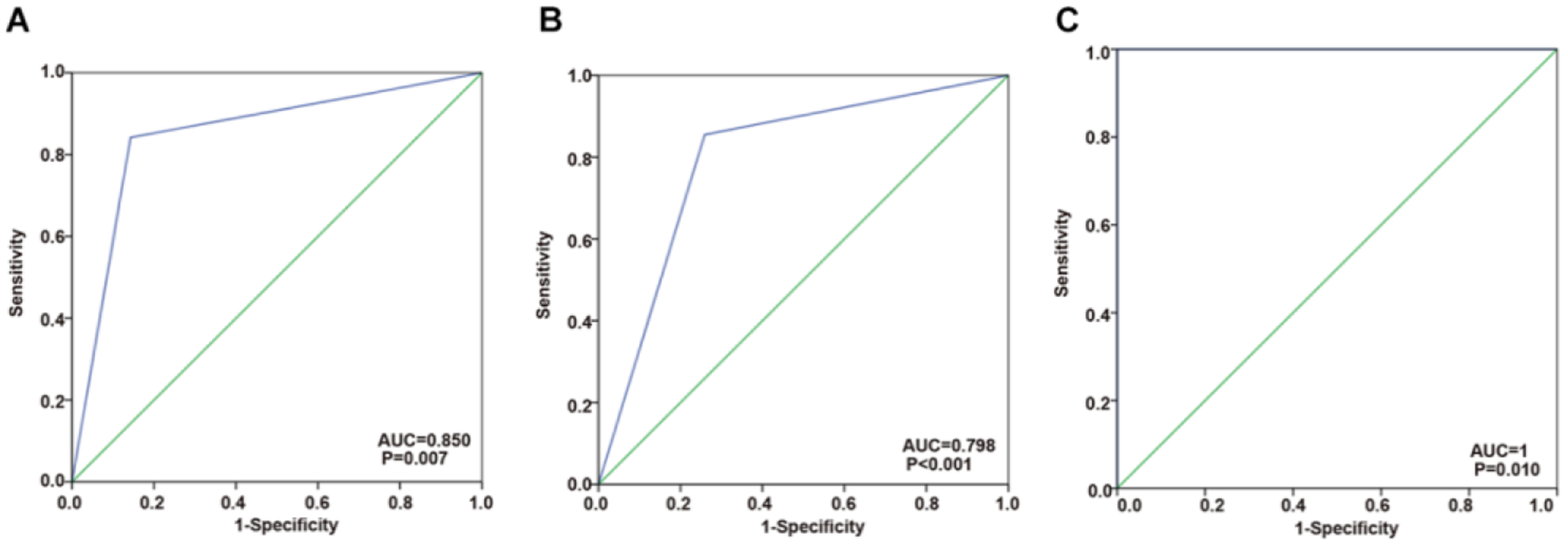


**Supplementary Figure 2. Receiver operating characteristics analysis of genetically abnormal cells for non-small cell lung cancer according to the nodule type. (A)** Pure ground glass type. Using a cut-off value of ≥3 CAC achieved 82.0% sensitivity, 77.8% specificity, the area under the curve (AUC) of 0.799, 96.2% positive predictive value (PPV), 38.9% negative predictive value (NPV), and 3.69 positive likelihood ratio (PLR). **(B)** Solid. Using a cut-off value of ≥3 CAC achieved 90.4% sensitivity, 73.9% specificity, AUC of 0.822, 91.7% PPV, 70.8% NPV, and 3.47 PLR. **(C)** Mixed. Using a cut-off value of ≥3 CAC achieved 85.3% sensitivity, 100% specificity, AUC of 0.926, 100% PPV, 50.0% NPV, and not-applicable PLR.


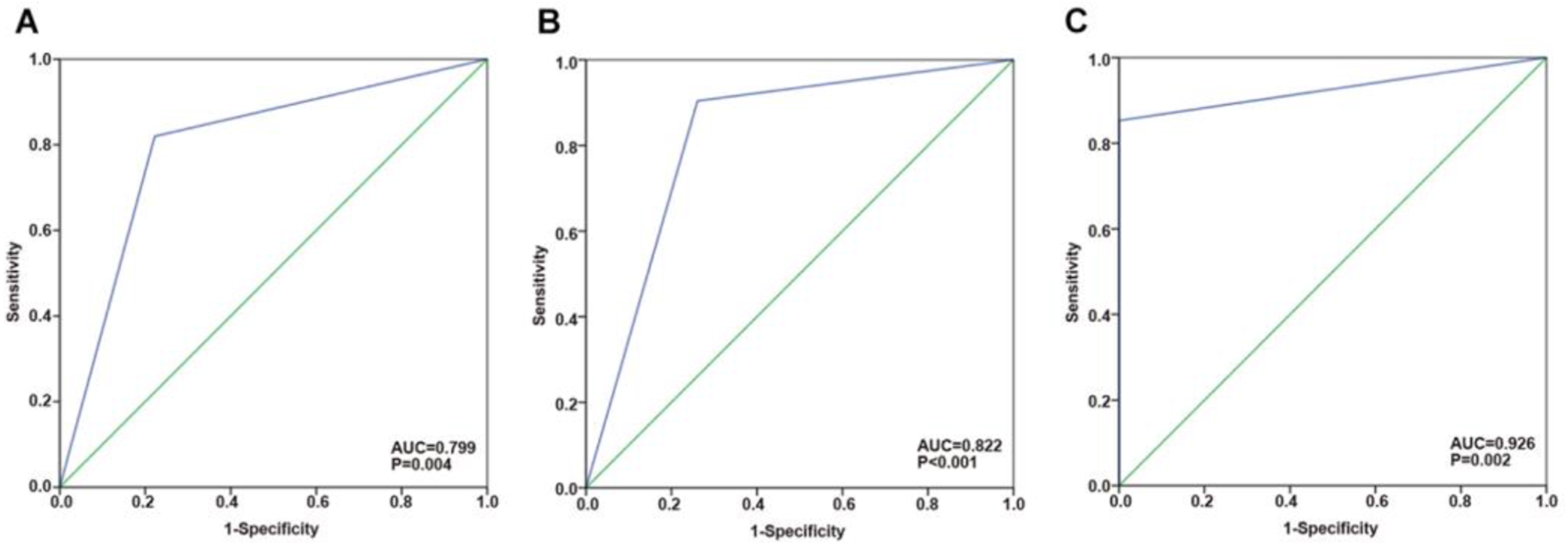

Supplement: Supplementary file 1 — (DOC 793 KB) [file 432_2021_3517_MOESM1_ESM.doc]
